# Experiences of intensive treatment for people with eating disorders: a systematic review and thematic synthesis

**DOI:** 10.1186/s40337-024-01061-5

**Published:** 2024-08-14

**Authors:** Hannah Webb, Maria Griffiths, Ulrike Schmidt

**Affiliations:** 1 Salomons Institute for Applied Psychology, Canterbury Christ Church University, Tunbridge Wells, TN1 2YG UK; 2 Centre for Research in Eating and Weight Disorders, Institute of Psychiatry, Psychology & Neuroscience, King’s College London, London, UK; 3 South London and Maudsley NHS Foundation Trust, Maudsley Hospital, Denmark Hill, London, SE5 8AZ UK

**Keywords:** Anorexia nervosa, bulimia nervosa, Eating disorders, Recovery, Intensive treatment, Qualitative research, Day patient, Inpatient, Patient perspective

## Abstract

**Background:**

Eating disorders are complex difficulties that impact the individual, their supporters and society. Increasing numbers are being admitted to intensive treatment settings (e.g., for inpatient treatment, day-patient treatment or acute medical treatment). The lived experience perspectives of what helps and hinders eating disorder recovery during intensive treatment is an emerging area of interest. This review aims to explore patients’ perspectives of what helps and hinders recovery in these contexts.

**Methods:**

A systematic review was conducted to identify studies using qualitative methods to explore patients’ experiences of intensive treatment for an eating disorder. Article quality was assessed using the Critical Appraisal Skill Programme (CASP) checklist and thematic synthesis was used to analyse the primary research and develop overarching analytical themes.

**Results:**

Thirty articles met inclusion criteria and were included in this review. The methodological quality was mostly good. Thematic synthesis generated six main themes; collaborative care supports recovery; a safe and terrifying environment; negotiating identity; supporting mind and body; the need for specialist support; and the value of close others. The included articles focused predominantly on specialist inpatient care and were from eight different countries. One clear limitation was that ethnicity data were not reported in 22 out of the 30 studies. When ethnicity data were reported, participants predominantly identified as white.

**Conclusions:**

This review identifies that a person-centred, biopsychosocial approach is necessary throughout all stages of eating disorder treatment, with support from a sufficiently resourced and adequately trained multidisciplinary team. Improving physical health remains fundamental to eating disorder recovery, though psychological support is also essential to understand what causes and maintains the eating disorder and to facilitate a shift away from an eating disorder dominated identity. Carers and peers who instil hope and offer empathy and validation are valuable additional sources of support. Future research should explore what works best for whom and why, evaluating patient and carer focused psychological interventions and dietetic support during intensive treatment. Future research should also explore the long-term effects of, at times, coercive and distressing treatment practices and determine how to mitigate against potential iatrogenic harm.

## Abstract (plain language summary)

Some people with eating disorders will need intensive treatment (e.g., inpatient treatment, day-patient treatment or acute medical treatment) during the course of their illness. Understanding what helps and hinders eating disorder recovery during intensive treatment is an important part of developing effective interventions. This review summarises research exploring people with eating disorders’ perspectives of intensive treatment, with the aim of identifying what helps and hinders eating disorder recovery. We searched in scientific databases for all published qualitative studies that explored people with eating disorders’ perspectives of intensive treatment. Thirty studies meet the inclusion criteria of this literature review. The results sections of these studies were analysed by extracting relevant findings relating to eating disorder recovery. We found that a person-centred, holistic approach is necessary throughout all stages of eating disorder treatment, with support from healthcare professionals and carers with specialist knowledge of how to support people with eating disorders. Improving physical health is fundamental to eating disorder recovery. However, psychological support is also essential to help people with eating disorders to understand what causes and maintains the eating disorder and support them to move away from an eating disorder dominated identity. Areas for future research are outlined.

## Introduction

Eating disorders (EDs) are a group of mental health disorders, such as anorexia nervosa (AN), bulimia nervosa (BN), and binge eating disorder (BED), that are characterised by severe disturbances of attitudes and behaviours related to food, weight, and shape, and that seriously impact mental and physical health [1]. ED onset is typically during late adolescence and early adulthood [2]. With the potential to impact every organ system, EDs can be life threatening, reportedly having the highest mortality rate of all mental health disorders [3–5]. EDs are burdensome to the individual, their supporters and society [6]. Covid-19 has only exacerbated this burden: increases in incidence rates, ED symptomatology and hospital admissions have been widely reported [7–9].

Treatment for people with eating disorders (PwEDs) depends on the severity and chronicity of difficulty [10]. Most PwEDs are first offered outpatient psychological therapy, which can be complemented with pharmacotherapy, medical monitoring, nursing and/or dietetic support [11]. For those who do not respond to outpatient treatment, or whose ED cannot be managed safely as an outpatient, intensive treatment may be offered. This typically ranges from day-patient treatment or partial hospitalisation to inpatient or residential treatment in an ED or general psychiatric unit. Though varied, these more intensive treatments typically involve greater multidisciplinary input and direct meal supervision [11]. Alongside specialist intensive treatments, increasing numbers of PwEDs are being admitted to general medical settings to manage the medical complications associated with EDs [12, 13]. Care in medical settings is highly variable, with varying levels of specialist input [11, 13]. Importantly, whilst the relative merits of each form of intensive treatment continue to be debated, demand appears to be rising internationally [14–16].

Clinicians supporting PwEDs encounter challenges due to the egosyntonic nature of the illness [17]. Many people attach positive value to their ED [18], as it gives a perceived sense of control, and means of obtaining identity and avoiding negative affect [19, 20]. Consequently, PwEDs are often ambivalent towards treatment and display low motivation to change [21, 22]. Current treatment efficacy is modest [23]. A recent rapid review suggested between 30% and 41% of PwEDs relapse within two years of receiving treatment and that less than half achieve recovery at long-term follow up [24]. Furthermore, across all EDs, 62–70% of people who have received inpatient treatment still meet full diagnostic criteria or have remaining ED symptoms at long-term follow-up [6].

To improve treatment outcomes for PwEDs, it is vital that we better understand the lived experiences of those who use ED services [25, 26]. As such, emerging research explores lived experience perspectives of ED treatment. For example, Babb and colleagues [27] reviewed qualitative studies exploring PwEDs’ general experiences of ED treatment. This review called for more individualised care and psychological support. Whilst valuable, it did not specifically focus on recovery. It also only identified studies exploring inpatient and outpatient experiences. Yet, some studies have explored PwEDs’ perspectives of other treatment settings, such as day-patient or acute medical settings, which may add important insights. The lifespan approach taken in this review may also mean that a review focused on adult populations is warranted as there are differences in ED treatment accessibility and delivery between child, adolescent and adult services. For instance, the duration of untreated ED (DUED) varies strongly between age groups, with younger age groups seeing shorter DUEDs [28] and in child and adolescent ED treatment, greater emphasis is placed on family involvement [29].

Other reviews seek to conceptualise ED recovery from lived experience perspectives. These have led to recovery being described as a complex psychological process that requires commitment, responsibility, development of insight into the function and consequences of the ED, acceptance by others and of the self, and development of meaningful relationships [30]. Recovery has also been said to include remission of ED symptoms alongside psychological well-being and adaptability, and involves hope, reclaiming identity, meaning and purpose, empowerment and self-compassion as key components [31–33]. Whilst valuable findings, these reviews do not focus specifically on what aspects of treatment help or hinder recovery.

More recently, two qualitative reviews synthesised literature exploring the lived experiences of inpatient treatment for all EDs [34] and AN only [35] within ED-specific treatment settings. These reviews highlight the complex and multifaceted nature of inpatient experiences and the importance of person-centred treatment that involves medical and psychological intervention [34, 35]. Undeniably, these reviews provide insight into a neglected area of research. However, they include differing all-age studies and exclude studies exploring different intensities and aspects of intensive treatment (such as the experience of involuntary admission). Yet, many PwEDs move through different intensive treatments, some outside ED-specific treatment settings, and all aspects of intensive treatment may relate to recovery.

ED recovery is a process rather than a singular event, which can begin before and continue beyond inpatient treatment. Therefore, this review aims to extend previous reviews exploring the lived experiences of inpatient treatment. With a focus on recovery, it aims to elucidate what helps and hinders recovery for adults with EDs across all types and aspects of intensive treatment and to provide recommendations for research and clinical practice.

## Methods

### Search strategy

This systematic review was conducted in line with Preferred Reporting Items for Systematic Reviews and Meta-Analyses (PRISMA) guidelines [36] and was pre-registered on PROSPERO (ID: CRD42023426052).

Systematic literature searches were carried out using electronic databases (EMBASE, MEDLINE, PsychINFO, and Web of Science), searched from conception to 6th June 2023. Search terms and inclusion and exclusion criteria were formed using the ‘Sample, Phenomenon of Interest, Design, Evaluation and Research type’ (SPIDER) tool [37], outlined in Table 1. The search strategy employed was informed by preliminary internet searches and previous reviews. It covered four concepts: [1] EDs, [2] intensive treatment, [3] qualitative methodology, and [4] lived experiences. Various combinations of search terms were trialled before settling on a broad search strategy that explored all free text to maximise search sensitivity.

**Table 1:** SPIDER search terms and Boolean operators

Sample AND	Phenomenon Of Interest AND	Design AND	Evaluation	Research Type
eating disorder* OR anore* OR bulimi* OR binge* OR EDNOS OR OSFED OR ARFID	inpatient OR IP OR intensive OR admission OR eating disorder unit* OR acute OR day patient OR day treatment OR day hospital* OR partial hospital*	qual* OR mixed method* OR case study OR content analysis OR discourse analysis OR ethnography OR exploratory OR focus group OR grounded theory OR interview* OR narrative OR phenomenology OR phenomenological OR thematic analysis	experience* OR attitude* OR perspective* OR view* OR reflect* OR interview*	N/A

Note. EDNOS = Eating Disorder Not Otherwise Specified; OSFED = Other Specified Feeding and Eating Disorder; ARFID = Avoidant Restrictive Food Intake Disorder

### Study selection and eligibility criteria

The first author completed the literature search, which yielded 2590 articles. Duplicates were removed, and the titles and abstracts of the remaining articles were screened against predetermined inclusion and exclusion criteria, outlined in Table 2. Qualitative or mixed method studies (if qualitative results were reported separately) that explored adults’ experiences or views of any aspect of intensive treatment directly related to an eating disorder diagnosis were considered for eligibility. Only studies originally published in English and in peer-reviewed journals were accepted. A decision was made not to search the grey literature due to time constraints and wanting to ensure adequate space and consideration was given to the included studies. Further, grey literature studies are not necessarily subject to the same rigorous academic peer-review processes as non-grey literature studies. Nonetheless, some potentially relevant studies may have been missed.

**Table 2:** Inclusion and exclusion criteria

	Inclusion criteria	Exclusion criteria
Sample	Focus on adults’ experiences of intensive treatment related to an eating disorder diagnosis. E.g., anorexia nervosa (AN), bulimia nervosa (BN), binge eating disorder (BED), eating disorder not otherwise specified (EDNOS), other specified feeding and eating disorder (OSFED), and avoidant restrictive food intake disorder (ARFID).	Focus on individuals with lived experiences of intensive treatment related to another mental or physical health difficulty, on individuals with lived experiences of only outpatient treatment for an eating disorder, or on child and/or adolescent eating disorder samples.
Phenomenon of Interest	Focus on the experience of current or past intensive treatment directly related to an eating disorder diagnosis. E.g., specialist eating disorder or general psychiatric inpatient treatment, day-patient treatment, partial hospitalisation, intensive community treatment or general medical admissions for eating disorder symptoms.	Focus solely on carers’ experiences or healthcare professionals’ experiences of intensive treatment for an eating disorder.
Design	Qualitative methodology (or mixed methods methodology, if qualitative results are reported separately) and used a named, bona fide analytic approach.	Quantitative methodology.
Evaluation	Explicitly attempt to capture individuals’ experiences, attitudes, perspectives, or views of any aspect of intensive treatment (e.g., overall experience, experience of an intervention, or exploration of a process).	Studies in which the qualitative data is minimal (e.g., no data extracts provided).
Research Type	Studies published in English. Studies published in peer-reviewed journals.	Studies published not in English. Grey literature studies.

**Fig. 1:**
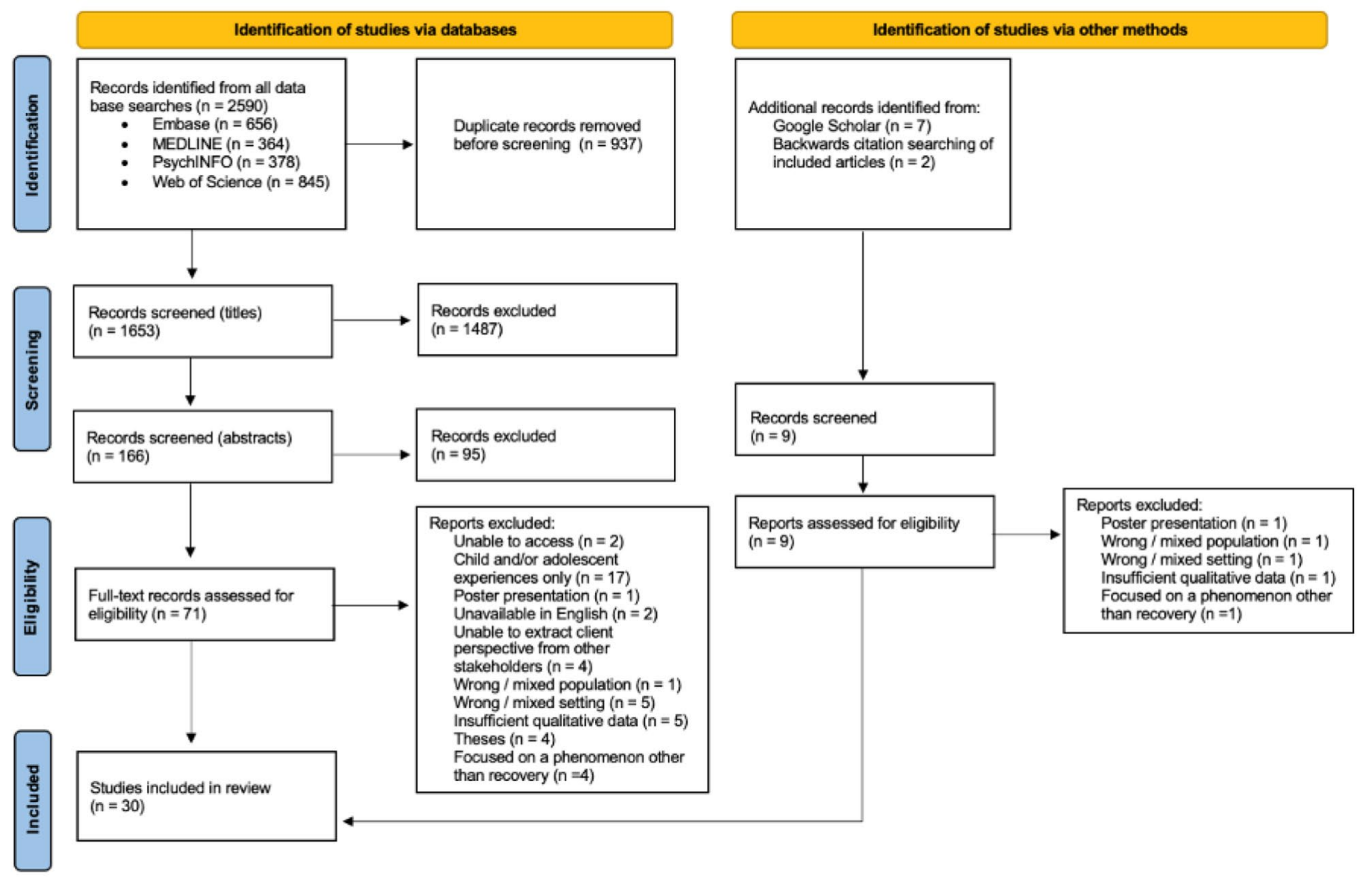
PRISMA Flow Diagram

Eligibility screening resulted in 71 articles which were read in full. Full-text screening excluded a further 45 articles, resulting in a total of 26 articles. The first author also screened the reference lists of included manuscripts to identify other studies that may have met the inclusion criteria and conducted additional searches through Google Scholar throughout the review process. This resulted in an additional four articles, meaning that 30 articles were included in this review. Throughout this process, any discrepancies were discussed with the second author (MG) until a consensus was reached. The complete procedure is detailed in the PRISMA diagram (Fig. 1).

### Quality assessment

Though what constitutes “validity” or “quality” in qualitative research is debated, quality appraisal remains a crucial part of any qualitative review [38]. The Critical Appraisal Skill Programme (CASP) checklist, a commonly used research appraisal tool, offers ten questions that facilitate assessment of qualitative studies. The Cochrane Qualitative and Implementation Methods Group recommends to avoid providing numerical scores, as CASP is not recommended as an absolute score of quality [39]. Instead, studies are considered according to whether criteria are: “yes well addressed”; “can’t tell”; or “no not addressed”. In this review, “can’t tell” was chosen when insufficient information was reported to make a judgement, as quality issues may be due to poor methodology and/or inadequate reporting [40, 41]. The first author conducted the quality assessment and any ambiguities were discussed with the review team until a consensus was reached.

Given the large number of studies in this review, whilst absolute scores were avoided, quality appraisal was used to organise the thematic synthesis, as has been recommended previously [e.g., 41, 42]. This meant studies (*n* = 10) for which “yes” was chosen for all ten questions were first reviewed to generate the coding framework. This was used to code the remaining studies. When particularly meaningful, new codes were generated. No studies were deemed to be low quality, as all studies provided valuable contributions to a limited evidence base. If there had been low quality studies, no new codes would have been generated, though these studies would not have been excluded.

### Method of synthesis

Thematic synthesis was chosen to integrate findings of multiple qualitative studies to answer a specific review question and extend what is already known [43]. All text from “results” or “findings” sections, and any findings in abstracts, were extracted and treated as data. Thematic synthesis followed three iterative stages. Stage one involved line-by-line coding of text according to meaning and content. Stage two involved grouping of codes into hierarchical structures, to develop descriptive themes that remained data-driven and close to the primary studies. Stage three involved the generation of analytical themes through inference of descriptive themes, which go beyond the primary studies to generate new interpretive explanations.

### Reflexivity

Reflexivity, the conscious, collaborative appraisal and critique of how one’s subjectivity and context influence the research processes, is an essential component of qualitative research [44, 45]. We, the three authors, have psychology/psychiatry and academic and clinical backgrounds. The first author is a trainee clinical psychologist with lived experience of an ED as well as academic and clinical experience in EDs/mental health. The second author is a clinical psychologist with academic and clinical experience in mental health, in particular with adults with experiences of psychosis. The third author is a consultant psychiatrist and expert in the field of EDs, with experience of developing national and international initiatives to improve ED policy and practice. One of us was an insider to the experience of ED treatment and we are all insiders to a culture of working in mental health services with often high levels of need and limited resource. We made every attempt to ensure potential biases (e.g., our combined clinical, academic and experiential understanding that intensive treatment can be challenging for many) were kept in awareness and endeavored to pay attention to the full range of findings. Coding extracts and theme developments were discussed with all authors to check for disagreements or uncertainties before being finalised. Additionally, the first and second author met for monthly supervision to discuss the review development and analysis, and to support a continuous process of self-reflection. This collaborative approach supported development of themes that captured important nuances in the lived experiences of ED treatment, for example identifying the tension between physical versus psychological support. Nonetheless, as with all qualitative research, a different group of researchers who sought to answer the same research question may have extracted different themes from the data.

## Results

### Studies identified

Thirty papers were identified as relevant. These are summarised in Table 3.

**Table 3:** Included study characteristics

Author(s), Year and Location	Aim(s)	Sample Characteristics: size (*N*), age, gender, ethnicity, diagnosis (Dx)	Treatment Setting, Length of Stay (LoS)	Recruitment	Data Analysis and Data Collection
Biddiscombe et al., 2018 Australia	To explore the experience of occupational therapy food groups in supporting functional recovery in an adult ED day program.	*N* = 99 Age: 26 (17–41) Gender: 98% F, 1% M Ethnicity: NR Dx: 11% AN, 30% BN, 3% BED, 45% EDNOS, Missing 10%	Setting: Specialist day-patient (4 days / week) LoS: 0.14 – 27 weeks	Invitation to past patients upon discharge from day program during a specified period Open ended questions (discharge and follow up questionnaires)	Thematic Analysis (adopting an inductive approach)
Clark Bryan et al., 2022 United Kingdom	To explore the process of transitioning from intensive treatment to the community.	*N* = 11 Age: 24.8 (16.8-32.8) Gender: 87% F, 13% M Ethnicity: 100% White Dx: 100% AN	Setting: Specialist inpatient and day-patient (various) LoS: NR	Invitation to past patients post discharge from intensive treatment during a specified period Semi-structured interviews	Thematic Analysis (adopting an inductive approach)
Cockell et al., 2004 Canada	To identify factors that help or hinder the maintenance of change and the promotion of recovery during the 6 months following ED treatment.	*N* = 32 Age: 27.9 (17.8-38) Gender: 100% F Ethnicity: NR Dx: NR	Setting: Specialist inpatient (15-week program) LoS: NR	Invitation to past patients 6 months post discharge from inpatient treatment Semi-structured interviews	Grounded Theory
Eli, 2014 Israel	To explore the experiences of specialist ED inpatient treatment in Israel.	*N* = 13 Age: NR (18–38) Gender: 92.3% F, 7.7% M Ethnicity: NR Dx: 92.3% AN, 7.7% BN	Setting: Specialist inpatient LoS: NR “considerable variations”	Invitation to past patients through various sources years after admission, as part of a longitudinal anthropology study Semi-structured interviews	Interpretative Phenomenological Analysis
Federici & Kaplan, 2008 Canada	To explore views of relapse and recovery in the first year following intensive treatment.	*N* = 15 Age: 26 (19.5-32.5) Gender: 100% F Ethnicity: 100% White Dx: 100% AN	Setting: Specialist day-patient and inpatient LoS: NR	Invitation one year following discharge to past patients with discharge BMI ≥ 20 Semi-structured interviews	Phenomenological Approach
Fox & Diab, 2015 United Kingdom	To explore experiences of living with and being treated for chronic AN in an inpatient treatment setting.	*N* = 6 Age: 27 (19–50) Gender: 100% F Ethnicity: 100% White British Dx: 100% AN	Setting: Specialist inpatient LoS: 4-27 months	Invitation to current inpatients during a specified period Semi-structured interviews	Interpretative Phenomenological Analysis
Hannon et al., 2017 United Kingdom	To explore experiences of long term intensive community treatment for individuals with severe AN.	*N* = 5 Age: NR (23–30) Gender: 100% F Ethnicity: 100% White British Dx: 100% AN	Setting: Intensive community treatment LoS: NR	Invitation to current and past patients who had received a full package of treatment during a specified period Semi-structured interviews	Interpretative Phenomenological Analysis
Hedlund & Landgren, 2017 Sweden	To elucidate experiences of receiving acupuncture as a complement to treatment as usual in inpatient treatment.	*N* = 9 Age: 30 (22–55) Gender: 100% F Ethnicity: NR Dx: 100% AN	Setting: Specialist inpatient LoS: NR	Invitation to current patients receiving acupuncture during a specified period Narrative interviews	Phenomenological Hermeneutic Method
Holmes et al., 2021 United Kingdom	To explore the experience of trust in inpatient treatment.	*N* = 14 Age: NR (20–42) Gender: 100% F Ethnicity: 78.6% White British, 21.4% White Jewish, 7.1% White American, 7.1% Other Dx: 100% AN	Setting: Specialist inpatient (various) LoS: NR	Invitation to past patients through an ED charity website and social media Semi-structured interviews	Thematic Analysis (within a poststructural, discourse-analytic framework)
İnce et al., 2023 United Kingdom	To explore the intensive treatment experiences of individuals with severe AN and their carers.	*N* = 6 Age: NR Gender: 100% F Ethnicity: NR Dx: 100% AN	Setting: Specialist inpatient and day-patient (various) LoS: NR	Invitation to past patients via email after routine 6-month follow-up assessment questionnaire completion Semi-structured interviews	Reflexive Thematic Analysis
Larsson et al., 2018 United Kingdom	To explore experiences of a perfectionism group intervention during inpatient treatment.	*N* = 14 Age: 27.4 (19.7-35.1) Gender: 100% F Ethnicity: NR Dx: 100% AN	Setting: Specialist inpatient LoS: NR	Invitation to current patients following completion of perfectionism group during a specified period Focus groups	Thematic Analysis
Long et al., 2011 United Kingdom	To investigate inpatient perceptions of mealtimes on specialist ED units.	*N* = 12 Age: 22.1 (17.4-29.5) Gender: 100% F Ethnicity: NR Dx: 100% AN	Setting: Specialist inpatient (various) LoS: NR	Invitation to current inpatients from ED units (three public, one independent) during a specified period Semi-structured interviews	Thematic Analysis
Mac Donald et al., 2023 Denmark	To explore experiences of patients with AN who have experienced multiple involuntary treatment events.	*N* = 7 Age: NR (states 20–30 s) Gender: 100% F Ethnicity: NR Dx: 100% AN	Setting: NR LoS: NR	Invitation to past patients of specialised units, the Danish patient organization, and the Danish Society for EDs (via flyers, websites, social media) Semi-structured interviews	Reflexive Thematic Analysis (adopting an inductive approach)
Matthews et al., 2019 Australia	To examine development and implementation of a day treatment program from patient and provider perspectives.	*N* = 11 Age: NR (17–33) Gender: 100% F Ethnicity: NR Dx: 81.8% AN, 18.1% Missing	Setting: Specialist day-patient LoS: NR	Invitation to all patients attending the day patient program during a specified period Semi-structured interviews	Framework Method
Matthews-Rensch et al., 2023 Australia	To describe the acceptability of a 7-day nasogastric refeeding protocol with adults with medically unstable EDs and staff involved in their treatment.	*N* = 8 Age: 22 (18–27) Gender: 100% F Ethnicity: NR Dx: 75% AN, 25% OSFED	Setting: Acute – medical stabilisation LoS: NR	Invitation to all participants undergoing refeeding during a specified period Semi-structured interviews	Framework Method
Money et al., 2011 United Kingdom	To explore patients’ experiences of CREST (Cognitive Remediation and Emotion Skills Training) during inpatient treatment.	*N* = 28 Age: 25 (13–40) Gender: 96.4% F, 3.6% M Ethnicity: NR Dx: 100% AN	Setting: Specialist inpatient LoS: NR	Invitation to current patients who had completed CREST during inpatient treatment during a specified period Open ended questions, as part of end of therapy reflection form.	Content Analysis
O’Connell, 2023 United Kingdom	To examine one individual’s lived experience of the diagnosis and treatment of anorexia nervosa.	*N* = 1 Age: NR Gender: 100% F Ethnicity: NR Dx: 100% AN	Setting: Specialist inpatient (various) LoS: NR (study relates to four admissions)	Not applicable. Personal diaries and community and hospital medical records.	Autoethnography
Pemberton & Fox, 2013 United Kingdom	To understand factors important in the care and emotional management of EDs in inpatient treatment.	*N* = 8 Age: NR (states that 7 were under 25) Gender: 87.5% F, 12.5% M Ethnicity: NR Dx: 100% AN	Setting: Specialist inpatient (two, one also with an intensive care unit) LoS: 0.5 – 6 months	Invitation to current patients of two inpatient units during a specified period Semi-structured interviews	Grounded Theory
Rienecke et al., 2023 United States of America	To understand the patients’ perspectives of involuntary treatment in an acute medical stabilisation unit.	*N* = 30 Age: 30.8 (20–54) Gender: 87% F, 10% M, 3% non-binary Ethnicity: 100% White Dx: 100% AN	Setting: Acute – medical stabilisation LoS: 7-74 days	Invitation to past patients who had been admitted involuntarily to the acute medical stabilisation unit. Semi-structured interviews	Thematic Analysis
Ross & Green, 2011 United Kingdom	To consider whether inpatient admission was a therapeutic experience for two women with AN.	*N* = 2 Age: NR (states both >18) Gender: 100% F Ethnicity: NR Dx: 100% AN	Setting: Specialist IP inpatient LoS: NR	Invitation to current patients during a specific period Semi-structured interviews	Thematic Narrative Analysis
Seed et al., 2016 United Kingdom	To explore how people with AN experience detention under the Mental Health Act and how these experiences impact on recovery.	*N* = 12 Age: 28.1 (18–43) Gender: 100% F Ethnicity: NR Dx: 100% AN	Setting: Specialist inpatient (various) LoS: NR	Invitation to current and past inpatients with experience of detention through one independent ED service, two National Health Service ED services, and an ED charity website Semi-structured interviews	Grounded Theory
Sly et al., 2014 United Kingdom	To examine the experiences of developing therapeutic alliance during inpatient treatment for an ED.	*N* = 8 Age: 25 (18–34) Gender: 100% F Ethnicity: 100% White Dx: 100% AN	Setting: Specialist inpatient LoS: NR	Invitation to current inpatients during a specific period Semi-structured interviews	Interpretative Phenomenological Analysis
Smith et al., 2016 United Kingdom	To explore the experiences of women currently undergoing specialist inpatient treatment for AN.	*N* = 21 Age: 25.2 (18–41) Gender: 100% F Ethnicity: NR Dx: 100% AN	Setting: Specialist inpatient LoS: 2-28 weeks	Invitation to current inpatients through a patient community meeting Semi-structured interviews	Thematic Analysis (adopting a realist and inductive approach)
Solhaug & Alsaker, 2021 Norway	To explore how patients with severe EDs experience inpatient treatment, and how they value the impact of their experiences in treatment.	*N* = 3 Age: NR (18–30) Gender: NR Ethnicity: NR Dx: NR	Setting: Specialist inpatient LoS: NR	Invitation to current inpatients during a specified period Diary entries	Thematic Analysis (adopting an interpretive approach)
Strand et al., 2017 Sweden	To explore patients’ experiences of participating in a self-admission program at a specialist ED unit.	*N* = 16 Age: 31 (18–56) Gender: 94% F, 6% M Ethnicity: NR Dx: 100% AN	Setting: Specialist inpatient LoS: NR	Invitation to current and past inpatients enrolled in a self-admission program Semi-structured interviews	Content Analysis
Whitney et al., 2008 United Kingdom	To explore service users’ experiences and perspectives towards receiving CRT (Cognitive Remediation Therapy) during inpatient treatment.	*N* = 19 Age: 30.3 (17–54) Gender: 100% F Ethnicity: NR Dx: 100% AN	Setting: Specialist inpatient LoS: NR	Invitation for feedback from current patients receiving CRT during their penultimate CRT session. Feedback letters	Grounded Theory
Williams et al., 2020 Canada	To explore the characteristics, outcomes and experiences of young adults accessing residential ED treatment.	*N* = 39 Age: 20.2 (18–24) Gender: 97.4% F, 2.6% non-binary Ethnicity: NR Dx: NR	Setting: Specialist inpatient LoS: 1-4.6 months	Invitation to current inpatients during a specified period Semi-structured interviews	Thematic Analysis
Wright & Hacking, 2012 United Kingdom	To explore the lived experience of therapeutic relationships between women with AN and their healthcare professionals.	*N* = 6 Age: NR (21–44) Gender: 100% F Ethnicity: 100% White British Dx:100% AN	Setting: Specialist day-patient LoS: NR	Invitation to current day-patients and their healthcare professionals during a specified period Semi-structured interviews	Thematic Analysis
Yim et al., 2023 United Kingdom	To explore patients’ experiences and perceptions of the utility of clinical team meetings (ward rounds)	*N* = 6 Age: NR Gender: 100% F Ethnicity: NR Dx: NR	Setting: Specialist inpatient LoS: NR	Invitation to current inpatients during a specified period Focus groups and one semi-structured interview	Reflexive Thematic Analysis
Zugai et al., 2018 Australia	To understand the nature of therapeutic alliance between nurses and patients with AN in inpatient treatment.	*N* = 34 Age: 20 (NR) Gender: 97% F, 3% M Ethnicity: NR Dx: 100% AN	Setting: Mixture of specialist ED and general mental health inpatient units LoS: NR	Invitation to current inpatients with experience of at least one week of treatment in one of six inpatient wards. Semi-structured interviews	Thematic Analysis (adopting a deductive and inductive approach)

Note. BMI = Body Mass Index; NR = Not Reported; LoS = Length of stay; F = Female; M = Male; AN = Anorexia Nervosa; BN = Bulimia Nervosa; BED = Binge Eating Disorder; EDNOS = Eating Disorder Not Otherwise Specified; OSFED = Other Specified Feeding and Eating Disorder

Included studies totalled 495 participants ranging from 17 to 56 years. 96% identified as female, 2% identified as male, 0.4% identified as non-binary and 0.6% were not reported. 65% of participants were diagnosed with AN, 6.3% with BN, 0.6% with BED, 9.1% with EDNOS, 0.4% with OSFED, and 18.6% as missing or not reported. Ethnicity data were not reported in 22 studies. When ethnicity data were reported, 98.9% of participants identified as white (94/95 participants in reporting studies) and 1% identified as Other.

Included studies were predominantly conducted in the United Kingdom (*N* = 17). Other countries included Australia (*N* = 4), Canada (*N* = 3), Sweden (*N* = 2), Denmark (*N* = 1), Israel (*N* = 1), Norway (*N* = 1) and the USA (*N* = 1). Most studies focused on specialist inpatient units only (*N* = 19), with three studies focusing on inpatient and day-patient settings and one study focusing on inpatient and general psychiatric units. Three studies focused on day-patient settings only and two studies focused on medical settings only. One study focused on intensive community treatment and one study did not report the setting (though it focused on experiences in intensive settings). Most (27/30) studies did not report length of stay and those that did reported a wide range of 0.14 to 27 months.

Recruitment was carried out using various methods, inviting both current and past receivers of treatment. A range of data analysis approaches were used, though half of the studies used thematic analysis. Most studies (*N* = 23) used semi-structured interviews. Other data collection methods included open-ended questions in discharge/feedback questionnaires, narrative interviews, focus groups, diary entries and medical documents.

### Quality appraisal

Included studies were of variable quality, but none were considered inadequate (see Table 4). All studies provided clear statements of the aims and appropriateness of qualitative methodology. The research design was unclear in three studies [46–48] and one study [49] did not explain consideration of ethics. Ten studies did not describe their recruitment strategy and thirteen studies did not provide any/adequate consideration of the relationship between the researcher(s) and participants. This contrasted with many studies that provided clear descriptions of their recruitment strategy (e.g., [50, 51]) and researcher reflexivity (e.g., [52, 53]). In line with their study methodology, some studies provided more descriptive analyses (e.g., [54, 55]) and others provided more in-depth analyses (e.g., [48, 49, 56]). Studies that did not provide sufficient qualitative data for the quality of their analysis to be considered and analysed as part of this review were excluded at the point of screening. All studies showed sufficient rigour, providing clear statements of findings and situating these within the wider literature.

Studies varied significantly in the time-point of data collection (e.g., during treatment, immediately after, retrospectively or a combination), with only some reflecting on the chosen time-point(s). Most studies focused on experiences relating to specialist inpatient treatment and only some adequately described the treatment setting. Moreover, several studies did not provide key participant characteristics, samples were not representative and no study focused exclusively on any ED other than AN.

**Table 4:** CASP Quality Appraisal

Author	1)	2)	3)	4)	5)	6)	7)	8)	9)	10)
Biddiscombe et al., 2018	Yes	Yes	Yes	Can’t tell	Yes	Can’t tell	Yes	Yes	Yes	Yes
Clark Bryan et al., 2022	Yes	Yes	Yes	Yes	Yes	Yes	Yes	Yes	Yes	Yes
Cockell et al., 2004	Yes	Yes	Yes	Can’t tell	Yes	Can’t tell	Yes	Yes	Yes	Yes
Eli, 2014	Yes	Yes	Yes	Yes	Yes	Can’t tell	Yes	Yes	Yes	Yes
Federici & Kaplan, 2008	Yes	Yes	Yes	Yes	Yes	Yes	Yes	Yes	Yes	Yes
Fox & Diab, 2015	Yes	Yes	Yes	Can’t tell	Yes	Yes	Can’t tell	Yes	Yes	Yes
Hannon et al., 2017	Yes	Yes	Yes	Yes	Yes	Yes	Yes	Yes	Yes	Yes
Hedlund & Landgren, 2017	Yes	Yes	Yes	Can’t tell	Yes	Yes	Yes	Yes	Yes	Yes
Holmes et al., 2021	Yes	Yes	Yes	Yes	Yes	Yes	Yes	Yes	Yes	Yes
İnce et al., 2023	Yes	Yes	Yes	Yes	Yes	Can’t tell	Yes	Yes	Yes	Yes
Larsson et al., 2018	Yes	Yes	Yes	Yes	Yes	Can’t tell	Yes	Yes	Yes	Yes
Long et al., 2011	Yes	Yes	No	Can’t tell	Yes	Can’t tell	Yes	Yes	Yes	Yes
MacDonald et al., 2023	Yes	Yes	No	Yes	Yes	Yes	Yes	Yes	Yes	Yes
Matthews et al., 2019	Yes	Yes	Yes	Can’t tell	Yes	Yes	Yes	Yes	Yes	Yes
Matthews-Rensch et al., 2023	Yes	Yes	Yes	Can’t tell	Yes	Yes	Yes	Yes	Yes	Yes
Money et al., 2011	Yes	Yes	Yes	Yes	Yes	Yes	Yes	Yes	Yes	Yes
O’Connell, 2023	Yes	Yes	Yes	Yes	Yes	Yes	Yes	Can’t tell	Yes	Yes
Pemberton & Fox, 2013	Yes	Yes	Yes	Yes	Yes	Yes	Yes	Can’t tell	Yes	Yes
Rienecke et al., 2023	Yes	Yes	Yes	Yes	Yes	Can’t tell	Yes	Yes	Yes	Yes
Ross & Green, 2011	Yes	Yes	Yes	Yes	Yes	Yes	Yes	Yes	Yes	Yes
Seed et al., 2016	Yes	Yes	Yes	Yes	Yes	Yes	Yes	Yes	Yes	Yes
Sly et al., 2014	Yes	Yes	Yes	Yes	Yes	Can’t tell	Yes	Yes	Yes	Yes
Smith et al., 2016	Yes	Yes	Yes	Yes	Yes	Yes	Yes	Yes	Yes	Yes
Solhaug & Alsaker, 2021	Yes	Yes	No	Can’t tell	Yes	Can’t tell	Yes	Yes	Yes	Yes
Strand et al., 2017	Yes	Yes	Yes	Yes	Yes	Yes	Yes	Yes	Yes	Yes
Whitney et al., 2008	Yes	Yes	Yes	Yes	Yes	Can’t tell	Yes	Yes	Yes	Yes
Williams et al., 2020	Yes	Yes	Yes	Yes	Yes	Can’t tell	Yes	Can’t tell	Yes	Yes
Wright & Hacking, 2012	Yes	Yes	Yes	Can’t tell	Yes	Can’t tell	Yes	Yes	Yes	Yes
Yim et al., 202Yes	Yes	Yes	Yes	Yes	Yes	Yes	Yes	Yes	Yes	Yes
Zugai et al., 2018	Yes	Yes	Yes	Can’t tell	Yes	Yes	Yes	Yes	Yes	Yes

Note. (1) = Clear statement of aims; (2) = Appropriate methodology; (3) = Appropriate research design; (4) Appropriate recruitment strategy; (5) Suitable data collection; (6) Adequate consideration of relationship between researcher and participants; (7) Consideration of ethical issues; (8) Rigorous data analysis; (9) Clear statement of findings; (10) Valuable research

### Thematic synthesis

Six themes were generated from the data: Collaborative Care Supports Recovery; A Safe and Terrifying Environment; Negotiating Identity; Supporting Mind and Body; The Need for Specialist Support; and The Value of Close Others. Themes and subthemes are outlined in Table 5 and discussed below.

**Table 5:** Themes and subthemes

Theme	Subthemes
Theme 1: Collaborative Care Supports Recovery	Active Involvement in Treatment Temporarily Handing Over Responsibility
Theme 2: A Safe and Terrifying Environment	A Bubble that was Hard to Replicate A Punitive, Distressing Environment
Theme 3: Negotiating Identity	Separating the Self and the ED Beginning to Want Something Different
Theme 4: Supporting Mind and Body	Weight Restoration and Dietary Change Psychological Awareness and Understanding
Theme 5: The Need for Specialist Support	Genuine Care, Alliance and Trust Skilled and Well Resourced Multidisciplinary Care
Theme 6: The Value of Close Others	Peer Support and Comparison Carer Support and Understanding Moving from Loneliness to Connection

### Theme 1: collaborative care supports recovery

#### Active involvement in treatment

Collaborative care supported recovery across intensive settings. “*Working together*” [51] and supporting PwEDs to “*make their own decisions*” [50] strengthened participants’ motivation. However, collaboration was “*often felt to be absent*” [54]. Several studies identified that participants felt “*alienated from the decision-making process*” [55], especially those admitted involuntarily. Feeling unheard negatively impacted upon self-esteem and anxiety. Lack of transparency between PwEDs and treatment providers affected treatment experiences and subsequent recovery. Lack of clarity about ward rounds led to *“power differences… and anxiety*” [57]. Participants in both studies exploring medical settings voiced not knowing who was chiefly responsible for their care and “*feeling deceived or given a punishment*” [55] when starting a refeeding protocol or being detained, due to lack of information. This negatively impacted upon treatment engagement. One study identified that providers should make expectations and regimes clearer and repeat them frequently “*to ensure patients have time to process and understand them*” [50]. In another study, the option to self-admit (to inpatient treatment) strengthened participants’ agency and motivation, and promoted partnership. However, for some, it risked too much decision-making power – “*too much say… it’ll be bad for me*” [56].

Collaboration was particularly key during transitions of care. Lack of information and “*uncertainty in what was going to happen*” [53] contributed to fear and feeling overwhelmed, hindering ongoing recovery. Many studies concurred that “*a graded and planned discharge helped…* [re]*integration*” [58]. This involved “*a phased*,* supportive approach*” [61], “*communication… with clear goals*” [54] and consideration of potential “*obstacles and challenges*” [63]. Several studies identified that treatment intensity dropped too quickly, that little or no further support was offered, or that participants were placed on lengthy outpatient waitlists. Continuity of support was essential.

#### Temporarily handing over responsibility

Whilst collaborative care generally supported recovery, there were instances in which, for short periods of time, participants found it helpful to not be so involved in care decisions. Several inpatient studies identified that, whilst challenging, many participants actually felt “*saved*” [58] when providers took responsibility (e.g., implementing clear boundaries around dietary change). “*Handing over”* [59] control was sometimes viewed as a necessary step towards recovery. However, for some, sudden loss of control contributed to heightened distress and “*amped up the ED*” [50]. For those experiencing involuntary treatment in particular (e.g., forced nasogastric feeding) this led to disconnection from one’s care. One study identified that “*hopelessness and resentment*” [58] developed. As Fox and Diab [49] outlined, the ED “*gave participants a sense of control and a method of coping*…” and “*refeeding… led to an intense feeling of losing control” –* supporting participants to understand the reasons behind care decisions and to process the intensive emotions these activated appeared fundamental to recovery.

### Theme 2: a safe and terrifying environment

#### A bubble that was hard to replicate

For some, the safety and security afforded by intensive treatment supported recovery. Inpatient and day-patient treatment granted “*permission*” [53, 58] to focus on recovery. Inpatients was described as a “*respite from overwhelming everyday demands*” [56]. Participants felt they “*belonged somewhere*” [64], finding “*comfort in predictable routines*” [65]. Inpatients also provided relief for carers. Several studies suggested non-negotiable boundaries supported change – “*completing meals was non-negotiable*” [66]. Two studies recognised when healthcare professionals (HCPs) made alterations to rules, it gave the ED “*leverage to pathologically negotiate*” [65]. Nonetheless, one participant identified that the existence of certain rules (e.g., prohibiting of water loading) alerted them to new possibilities.

It was recognised that the certainty and boundaries inpatients afforded was “*not easily replicated*” [52]. Their loss after discharge contributed to difficulties with continuing recovery. Indeed, inpatients was called a “*bubble*” [58, 59], “*greenhouse*” [60] and *“lab…* [with] *very exact and measured conditions*” [60]. It left participants “*frozen… and dependent on the unit*” [59]. Various studies identified that intensive treatment (particularly inpatient treatment) put “*life on hold*” [61]. For some, this contributed to dependence on treatment and the ED. As O’Connell [66] outlined, the ED became “*the standpoint from which I related to others*”. A few studies highlighted the importance of providers “*showcasing interest and highlighting aspects of patients’ lives outside of their ED*” [50] to provide relief from institutionalisation and support motivation. As PwEDs transitioned out of intensive treatment, returning to or beginning careers, relationships, leisure and personal development activities supported “*a sense of routine and purpose*” [61].

#### A punitive, distressing environment

Words such as “*miserable*”, “*horrific*”, “*hostile*”, “*traumatic*”, “*distressing*”, “*inhumane*”, “*terrifying*” and “*an assault*” were used to describe treatment (in inpatient and medical settings only) [48, 49, 54, 60, 64]. For some, feeling dehumanised, restricted or traumatised negatively impacted upon motivation, engagement and subsequent recovery. Several studies suggested participants felt “*under inspection*” [58] and treatment was described as “*doing time*” [67]. “*Exposure to…* [and experiences of] *distressing events*” [54] were difficult – described as “*something I’ll never forget*” [48]. Participants sometimes experienced “*corrective measures as punitive or disciplinary*” [65]. Moreover, across several studies, participants felt certain boundaries were arbitrary, employed without adequate explanation, or “*rigid and unable to be maintained*” [58], leaving them feeling disempowered.

### Theme 3: negotiating identity

#### Separating the self and the ED

Across many studies, attachment to the ED hindered recovery. The ED afforded safety, control and confidence in its success and provided “*emotional and physical detachment*” [62]. Intensive treatment “*created a state of internal coercion*” [48]. Several studies identified that a mismatch between treatment requirements and participants’ readiness to change could result in treatment refusal or termination, strengthening attachment to the ED. For those who experienced repeated admissions, lengthy stays or passing between services, “*feelings of hopelessness*” [49] and “*feelings of failure*” [56] were prevalent. Consequently, participants “*gripped more tightly onto AN*” [66] (and the ED identity).

Indeed, being “*reduced to a number and a disorder*” [55] in inpatient and medical settings hindered recovery. Various studies suggested participants disliked feeling defined by their illness and treated as “*a collective*” [60] or in accordance with “*an assumed group identity*” [68]. This *“one-size-fits-all approach*” [67] left participants feeling “*misunderstood*,* invalidated and stereotyped*” [66]. There was a desire for “*different tracks for people with different needs*” [55] and a wish for providers to “*humanise the patient*” [50]. Indeed, personalised, flexible treatment supported recovery across intensive settings. Day-patients was viewed as more flexible than inpatients, though both groups desired a more *“tailored approach*” [61] (e.g., better consideration of differences in sexuality, gender identity and comorbidities). Intensive community treatment was considered individualised, with “*specific and obtainable goals*” [62]. Moreover, several studies highlighted that, for some participants, being supported to externalise the ED as separate to their sense of self - recognising “*AN as pathology separate to who they were*” [65] - supported change and recovery.

#### Beginning to want something different

Indeed, ambivalence towards treatment, particularly initially, was common. Recovery required moving from ambivalence to acceptance and/or determination. Reflecting back, one participant suggested others should “*surrender a little bit*… *trust in the treatment*” [50]. For some, this was difficult. Several studies identified that compliance resulted in discharge, but not necessarily recovery. One participant “*humour*[ed]” [63] providers and another aimed to “*eat their way out*” [58]. It was these participants where relapse was most likely. Self-criticism, shame, worthlessness and hopelessness kept participants stuck.

Conversely, several studies outlined the value of motivation. In their study exploring experiences of recovered versus relapsed PwEDs, participants’ “*own drive*” [63] was prevalent in the recovered group. One participant described eventually “*wanting something different*” [66] and another study noted EDs require “*extremely hard work to be fought against*” [62]. Key to recovery was self-acceptance, hopefulness, and awareness and insight into the ED: “*compassion… and self-care*” [58] and “*a sense of self*” [64] were necessary.

### Theme 4: supporting mind and body

#### Weight restoration and Dietary Change

Many participants retrospectively saw intensive treatment as “*saving lives*” [48], specifically regarding medical stabilisation. However, across inpatient and medical settings, participants struggled with discrepancy between “*normal* [weight restored] *bodies*” and continued “*anorexic thoughts*” [63], leading to other maladaptive behaviours or relapse. Overfocus on biological markers, for example “*micro-monitoring of the participant’s weight*” [67], negatively impacted recovery. Across studies, participants wished for a “*slow pace of change with focus on all aspects of their difficulties*” [62].

Nonetheless, across specialist settings (i.e., not general medical), support in understanding and implementing dietary changes facilitated recovery. Meal support, plans and routines developed “*behavioural patterns that supported recovery*” [52] and “*staff eating alongside*” [46] normalised mealtimes. Nutritional education was also valued. Learning about “*daily nutritional requirements”* [52] and “*their bodies’ need for food*” [47] helped participants make dietary changes. Similarly, opportunities to engage in practical food groups (e.g., grocery shopping, outings to restaurants/cafes and meal preparation activities) were considered important and increased *“confidence to attempt repeating the challenges outside”* [69]. Practicing dietary related cognitive skills and coping strategies supported a “*gradual shift to more independent eating*” [70].

#### Psychological awareness and understanding

Understanding what caused and maintained the ED arose as integral to recovery, through individual and group therapy and wider psychological support. Individual therapy supported PwEDs to understand the ED and “*challenge… maladaptive thinking styles and behaviours*” [71]. A “*strong* [therapeutic] *connection*” [70] was essential. Similarly, a range of therapeutic groups, including Cognitive Behavioural Therapy, Dialectical Behavioural Therapy and the Maudsley Anorexia Nervosa Treatment for Adults groups, as well as perfectionism, mindfulness, and value-based groups, were appreciated. Many recognised *“the importance of sharing experiences and learning from each other”* [72], though for a minority, the perceived intensity of groups was challenging. A holistic therapy, acupuncture, was “*relaxing*,* both emotionally and physically*” [73] particularly after meals. Nonetheless, for some, therapy was “*too structured*” [74]. There was desire “*for more guidance and practice to help with real life application*” [71] and several studies identified a need for longer therapeutic intervention. One study identified insufficient psychological input in ward rounds, though one participant did not want their formulation shared due to it being “*very personal*” [57].

Learning to identify, express and manage emotions emerged as beneficial across intensive settings. For example, developing strategies to “*manage… and label emotions*” [74] and communicate one’s feelings supported recovery during and after treatment. Self-examination skills (e.g., journaling) helped PwEDs “*continue to work on recovery after discharge*” [52]. Several studies identified that emotional suppression and avoidance of negative affect limited progress.

### Theme 5: the need for specialist support

#### Genuine Care, Alliance and Trust

Genuine care, trust and therapeutic alliance between PwEDs and HCPs was important for recovery. Participants wished to be treated with dignity and respect. They valued HCPs who were “*approachable and friendly*” [51], empathic and non-judgemental, and who validated and managed participants’ emotions. For some, feeling cared for involved nurses adopting a “*motherly or sisterly role*” [65] and HCPs who went “*beyond their roles*” [54, 75]. Several studies noted the importance of strong therapeutic alliances with key workers, characterised by honesty, trust and openness. This promoted “*hope and optimism*” [75] and led participants to feel “*held or supported*” [62]. Without a good keyworker relationship “*challenges could feel insurmountable*” [51].

Correspondingly, across several studies, feeling uncared for negatively impacted recovery. Participants sometimes felt dismissed, patronised or ignored. They struggled with HCPs who “*failed to follow through with promises*” [58], “*overlooked* [them] *in comparison to newly admitted patients*” [59], or offered a *“lack of a predictable response”* [68]. Distrust between PwEDs and HCPs was “*an important precursor to some difficult interactions*” [67]. Described in several studies, conflict often led to further rebellion as the participant sought to “*retain their sense of control*” [46]. Poor connections resulted in increased anxiety and distrust, which impacted participants’ self-esteem, motivation, and desire to remain in treatment.

#### Skilled and well Resourced Multidisciplinary Care

Several studies outlined the importance of PwEDs being care for by a skilled and well resourced multidisciplinary team, with “*staff from different disciplines… contributing to residents’ recovery*” [70]. Changing teams, HCP shortages and use of non-permanent staff decreased standards of care and hindered recovery. Whereas, well trained and skilled HCPs displayed empathy, understanding, knowledge and clear boundaries. Indeed, “*trust and belief in practitioner’s expertise were… fundamentally important*” [49]. Skilled HCPs were able to separate the person from the ED, facilitate honesty and openness, and develop strong therapeutic alliances.

### Theme 6: the Value of Close others

#### Peer support and comparison

Peer support and comparison affected recovery. Across intensive settings, “*physical and behavioural comparisons*” [59] and competitiveness negatively affected *“group cohesion and personal recovery*” [53]. Many found it distressing and triggering being admitted alongside others at various stages of recovery and with differing levels of illness severity. Indeed, participants were susceptible to adopting *“new* [unhelpful] *ED practices*” [60]. Participants in two studies described comparing themselves (not under section) to those under section. This comparison increased participants’ guilt for choosing to eat and negatively impacted recovery. Correspondingly, participants in one study valued spending time with people without EDs who “*value aspects of life other than shape and weight*” [52].

In contrast, many of the same studies recognised that being alongside other PwEDs also supported recovery. Peers who understood and were non-judgmental were valued and contributed to connectedness, acceptance and belonging. Peer support “*increased knowledge of effective coping skills and hope for recovery*” [59]. Several studies noted participants made “*close and lasting friendships… through a sense of camaraderie*” [60]. Relatedly, one participant valued a peer mentor who had “*been there and got through*” [53].

#### Carer Support and understanding

Carer support and understanding during, and upon leaving, intensive treatment supported recovery. Across settings, participants desired for carers to “*provide love*,* a listening ear*” [50], particularly “*during the transition period*” [61]. Carer support groups were also valued. Returning home with “*insufficient or unhelpful social support*” [69], as well as “*continual emphasis on body weight and dieting within the family or social environment*” [63], hindered recovery.

#### Moving from loneliness to connection

Isolation hindered recovery. Particularly upon admission, participants described an emptiness, loneliness and difficulty trusting others. Difficulties developing and maintaining relationships contributed to negative attributions of the self and others and pushed participants further into their ED. Admissions sometimes exacerbated these difficulties as participants were removed from friends and family. Fostering “*meaningful connections after treatment*” [52] and moving from “*loneliness… to interpersonal connection*” [62] supported PwEDs to move towards recovery.

## Discussion

This review explored what helps and hinders recovery during intensive treatment for PwEDs. Participants acknowledged that intensive treatment was often necessary, particularly with regards to biomedical recovery. As higher discharge BMI predicts more positive outcomes (for AN) [76], promoting adequate weight restoration remains a priority. Nonetheless, consistent with existing literature [30, 35], a biomedical focus often took precedence over addressing underlying psychosocial difficulties. Participants were weight-restored but not recovered and often discharged without a period of consolidation or without adequate step-down support, placing them at higher risk of relapse following discharge [31]. Providers should be careful to not over-focus on biological markers and should ensure pace of change is acceptable to the individual.

Correspondingly, a therapeutic milieu, comprising individual and group therapy and the wider care environment, was valued and necessary for recovery, though was not always present or sufficient. Consistent with existing literature [77, 78], psychological interventions that supported PwEDs to understand the function and maintenance of their ED, as well as to identify, express and process emotions, facilitated recovery. Externalisation also arose as an important therapeutic technique across the wider care environment to foster separation from an illness identity [79, 80].

Ambivalence, resistance to change and hopelessness hindered recovery. Commonly identified as barriers to recovery [81–83], if these factors were not attended to, change was difficult, and relapse was likely. Imposing actions (e.g., through boundaries and routines) may be necessary for an individual’s safety, but carry a risk of driving them further into their ED, increasing resistance and decreasing motivation and compliance [84]. These findings support research highlighting the role of holding and actively sharing hope [33, 85] and of motivational interviewing [86].

Consistent dietary support should be embedded into intensive treatment. Across intensive settings (except in medical settings, where they were not mentioned), structured mealtimes, meal support, modelling normal eating, meal plans, nutritional education, and food groups supported PwEDs to move towards recovery. Supporting a small body of literature [87, 88], dietary-related interventions allowed PwEDs to practice adaptive coping strategies, improve eating behaviours and self-efficacy, and address social challenges associated with eating.

Compassionate and yet boundaried HCPs were essential. Across intensive settings, collaborative, person-centred care strengthened hope and engagement. PwEDs desired active involvement in treatment, though for some, having responsibility removed initially was a necessary part of recovery. As clinicians have highlighted, balancing PwEDs’ desires with beneficence can be challenging [85, 89], however the dominant medical paradigm, that positions HCPs as expert authorities, may harmfully limit choice, autonomy and opportunities for treatment participation. When PwEDs feel unheard or that their needs are not being met, premature treatment termination may result [90]. Whilst those in intensive settings are often at higher risk, where possible, it remains important to offer choice and clear information. Although few in number, studies exploring day-patient and intensive community settings suggested they afforded greater choice and collaboration, though this may be as these settings generally support less severe ED populations [91].

Experiences of care were highly individual. At times, intensive environments facilitated recovery. They were safe and supportive, due to firm boundaries, clear routines, and, in inpatient settings, escape from life stressors. Yet, consistent with ED clinicians’ concerns [85], intensive treatment (especially inpatient) also contributed to treatment dependence and estrangement from life outside. Transition out of intensive treatment was highlighted as a particularly vulnerable period. Day-patient and intensive community treatment discharges were experienced as somewhat more graded and skills learnt as more transferable, perhaps leading to a greater likelihood of maintenance. These findings underscore the value of intensive treatment but also the need for a gradual discharge process. Occupational therapists may be particularly well placed to support development of necessary skills for continuing recovery, supporting PwED’s to identify purpose outside of the ED, cope with external triggers and resume educational, vocational and/or family roles [87].

Intensive environments (in inpatient and medical settings only) were also experienced as restrictive and traumatising, due to experiences of coercion, scrutiny, and being subjected to, or witnessing of, distressing practices. These iatrogenic factors may hinder recovery and have long-lasting effects, contributing to more severe psychopathology and/or trauma-related symptoms. To date, limited work has explored what aspects render the experience of psychiatric hospitalisation distressing, though experiences of coercion, stress and trauma appear common and distressing [92]. Moreover, whilst compulsory treatment can be necessary to save lives, the long-term effects are largely unknown [93].

Adding to the growing literature base surrounding the value of carer support for adults with EDs [94, 95], carer support was valued when carers were able to understand the ED and challenges of treatment and offer empathy and validation. Given that carers’ distress and ways of coping can inadvertently maintain or reinforce the ED [96], this finding affirms the necessity for carers to receive their own support [95]. Currently, a range of carer interventions show positive outcomes for PwEDs undergoing intensive treatment, though implementation is patchy, and research has predominantly focused on young people with AN and the experiences of mothers [95].

Peer comparison, competition and contagion were common in intensive settings and often reinforced the ED-dominant identity. Nonetheless, peer support and identification were also common, and frequently decreased isolation while motivating individuals towards recovery. One study also highlighted the value of a peer mentor. As a growing area of research and clinical practice, peer mentors may instil hope and increase motivation for treatment [97]. Treatment alongside other PwEDs being both helpful and hindering for recovery is a widely reported juxtaposition [27, 85]. Helpful peer influence appears to depend on dis-identification with the ED-dominant identity and identification with a recovery identity. Indeed, a sense of shared identity with others in ED recovery promoted recovery in an online support group [98]. Specialist support is necessary and valued by PwEDs and this generally means PwEDs are treated alongside peers. Peer influence should therefore be considered as part of each individual’s formulation, to explore the potential for support and harm and how this may relate to the ED identity.

### Clinical and research implications

To enhance likelihood of ED recovery, a multidisciplinary approach is required across intensive settings. Restoring physical health remains fundamental. However, psychological support is also necessary. Whilst several psychological treatments have evidence supporting use in outpatients, minimal evidence guides implementation of evidence-based practices in intensive settings [99, 100]. Interventions that enhance motivation to change [86, 101], foster separation from an ED-dominant identity [102, 103] and support emotion recognition, regulation and expression [104, 105] should be prioritised. Research must determine what works best for whom and why, tailoring processes to PwEDs’ unique needs, contexts and goals [30] and comorbidities [106].

Specialist dietetic support should also be employed. Dieticians possess unique skills and knowledge, but the extent to which they are involved in intensive treatment is largely unknown [88] and limited research guides the content of dietetic interventions or explores the effect of including dietetics [107, 108]. Further research should explore what constitutes effective dietetic support across intensive settings [87, 108].

Time to consolidate recovery gains alongside planned and phased discharges are vital for ED recovery. Research has begun to explore novel ways to support intensive treatment transitions [109] and intensive stepped-care treatment programs highlight the value of longer-term multidisciplinary care for PwEDs [110, 111]. Further research must explore how to support maintenance of recovery, particularly as PwEDs return to daily life stressors.

Clinical practice guidelines recommend carer involvement in adult ED treatment [112, 113] and carers and PwEDs recognise the value of carer support [96, 114]. Current carer support is inconsistent, interventions vary, and a sufficient evidence base is lacking, particularly for adult ED populations [94, 115]. Carer capacity, skill and knowledge vary and interventions need to be tailored accordingly [95, 96]. To develop more routine and individualised care, research needs to elucidate which carer interventions works best for whom and why, taking consideration of different carer types, EDs other than AN, and stages of illness [94, 96].

Perhaps most notably, this review highlights the complexity of intensive support for PwEDs. Findings highlight several dilemmas that HCPs face: helpful boundaries and containment versus restriction and coercion; peer support versus contagion; and physical versus psychological recovery. There is a clear need for sufficient resource, specialist training and opportunities for HCPs to engage in reflective spaces. Organisational pressures alongside client complexity mean HCPs can find working with PwEDs emotionally draining, leading to negative judgements, frustration, hopelessness and worry [99, 116]. Perhaps it is these feelings that lead HCPs to strive for a practice of safe-certainty (e.g., administering standardised protocols) [116]. Time and space for reflection may support adoption of positions of safe-uncertainty, and consequently more flexible, person-centred approaches based on formulation and evidence-based interventions [116].

Specialist skills and knowledge, alongside trust and openness, reduce conflict and enhance therapeutic relationships and treatment engagement [117–119]. Within intensive settings, HCPs must balance firmness and empathy, communicating with clear boundaries to ensure certain behaviours are minimised whilst at the same time recognising and understanding the defensive nature of the ED and its adaptive function [22]. Future studies should explore what aspects of intensive treatment may be causing harm and any long-term effects. Moreover, there is need for specialist training and research in general medical settings, given the extent of negative experiences in this area.

#### Strengths and limitations

This review brings together 495 participants’ perspectives across thirty studies. Extending findings of previous reviews [34, 35], this study explores what helps and hinders recovery across the spectrum of intensive treatment specifically for adults with EDs. A rigorous methodological process was employed in the selection, evaluation and interpretation of studies. To ensure findings remained contextualised, details of each included article’s aims, sample, setting, methods and methodological quality were included. However, a number of limitations must also be considered. As grey literature was not searched, some potentially relevant studies may have been missed. However, the sample is purposive rather than exhaustive, as this review aims to offer interpretive explanation and not prediction, therefore it may not be necessary to locate every available study [43]. The majority of included studies explored inpatient treatment experiences. Whilst the number of studies exploring lived experiences in non-inpatient settings is limited, the included studies offer a glimpse into experiences of these settings and highlight an important research gap. Further research is needed into lived experiences of intensive treatment settings other than specialist inpatient treatment for PwEDs (e.g., exploring lived experiences of day-patient treatment/partial hospitalisation, residential care, intensive community treatment, home-based treatments and acute medical admissions). Moreover, many studies also inadequately described the treatment setting. Given the diversity of intensive treatment approaches for PwEDs, authors should endeavour to describe treatment settings adequately to support transferability of findings [120]. Additionally, included studies omitted several key participant characteristics, and as has been identified previously, samples lacked ethnic, gender and diagnostic diversity. This limits the generalisability of findings to groups other than white women with AN. Researchers must include ethnicity data, as its absence further maintains underrepresentation. Research prioritising the treatment experiences of marginalised groups is urgently required [121].

## Conclusions

This review explores what helps and hinders recovery during intensive treatment for PwEDs. A sufficiently resourced and adequately trained multidisciplinary service, which includes physical, psychological, dietetic and social support, supports ED recovery. Findings emphasised the vital role psychological support and understanding can have in supporting PwEDs to move from an ED-dominant identity to a sense of self outside of the illness and the value of carers and peers who instil hope and offer empathy and validation. Nonetheless, HCPs face several challenges when supporting PwEDs in intensive settings, as what is helpful for one person may be harmful for another. A person-centred, biopsychosocial approach is necessary throughout all stages of treatment. Further research must evaluate patient and carer focused psychological interventions and the role of dietetic support during intensive treatment. It must explore the long-term effects of, at times, coercive and distressing treatment practices and determine how to mitigate against potential iatrogenic harm.

## Data Availability

Data is provided within the manuscript. Further data is available on request.
